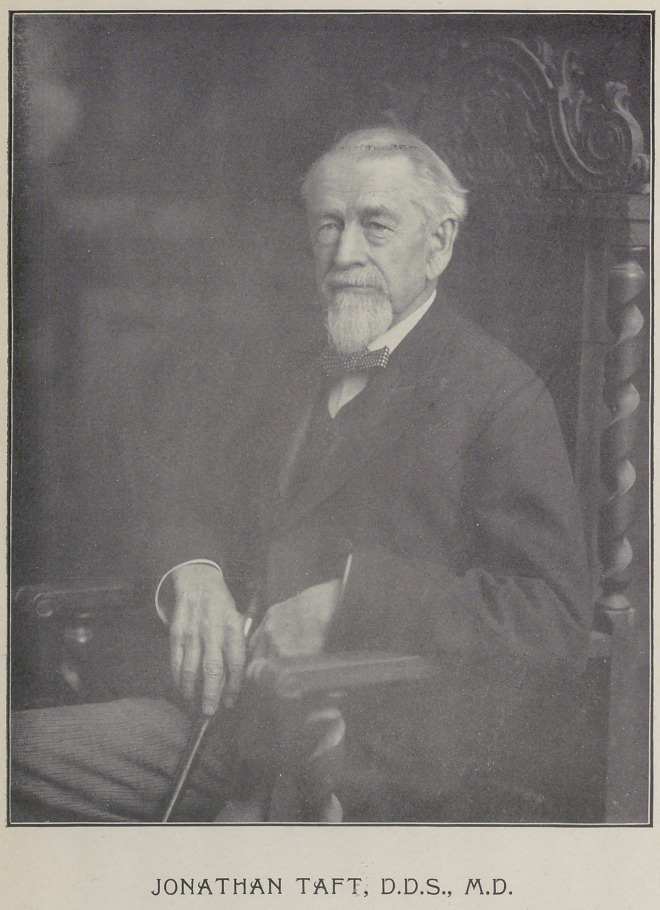# Jonathan Taft, D.D.S., M.D.

**Published:** 1903-11-15

**Authors:** 


THE
DENTAL REGISTER.

Vol. LVII. NOVEMBER 15, 1903. No. 11.

### JONATHAN TAFT, D.D.S., M.D.

Jonathan Taft was born September 17, 1820, in Russellville, Brown County, O., and died October 16, 1903. He was educated in the district schools and a small academy in Brown County, O. His father was a native of Massachusetts and a farmer by occupation; his mother was a native of Ohio. An injury, which was not properly treated, resulted in permanently crippling young Taft, incapacitating him for the hard work of the farm. He educated himself to teach school, which he did successfully for about four years.

In 1841 he took up the study of dentistry in Ripley, O., and began practicing for himself two years later in Ripley. A year afterward he moved to Xenia, O., and built up a good practice. It was while practicing here that he made the acquaintance of George Watt, and here began that long career of friendship and business relations which were kept up until Dr. Watt's death. The profession is greatly indebted to this friendship for much that it incidentally contributed to its advancement. They labored together on the DENTAL REGISTER with little or no compensation for the several years just preceding the war, and until 1864, when Dr. Watt resigned his place as associate editor to enter the army.

During a part of this time Dr. Taft's health was considered so precarious that it was feared he would go into a decline. Dr. Watt said once to the writer that he had spent many a wakeful hour on his bed conjuring up funny stories to relate to Dr. Taft to cheer him up and brighten his life.

Dr. Taft continued in regular practice for nearly sixty years, most of the time in Cincinnati. He rarely was ill and almost never took what is now popularly known as a vacation. He took his recreation while attending dental societies, and it is safe to say that he attended more dental society meetings than any man that ever lived. He gave up practice in 1901 and moved to Ann Arbor, Michigan, and gave all his time to college work.

In 1850 Dr. Taft graduated from the Ohio College of Dental Surgery, and in 1881 the University of Michigan conferred on him the honorary degree of Doctor of Medicine. In 1854 he was called to be Professor of Operative Dentistry in the Ohio College of Dental Surgery and a few years later was made its dean.

He retained this professorship until 1878 when he resigned to give his time more fully to a similar position he had accepted at the University of Michigan in 1875. The Ohio Dental College was the second dental college organized in the world, and when Dr. Taft came to it, he found that there was no suitable text-book on the subject of his lectures, and he wrote the first work—exclusively devoted to this subject. This book was a standard authority on this subject for nearly twenty-five years, as it ran through several editions. His work as editor of the DENTAL REGISTER was also of a pioneer character. The REGISTER was first published by the Mississippi Valley Dental Association ostensibly. but in reality Dr. James Taylor, of Cincinnati, was responsible editor and publisher for the first ten years of its existence. Then Dr. Taft and Dr. George Watt began the work which Dr. Watt resigned in 1864, and which Dr. Taft continued until 1900.

It is remarkable that this journal is now in its fifty-seventh year and has never lapsed for a single issue, making it the oldest dental journal in existence. Dr. Taft's wonderful energy and determination made this practicable. There is no doubt but that a less determined person would have long since given up the fight, as there were not a few periods in its history which were so critical as to threaten its existence. The REGISTER was the first dental journal to issue monthly. Very soon afterward the *News Letter* was dropped and succeeded by the *Cosmos*, which issued monthly. For many years the REGISTER was the organ of the whole country west of the Allegheny Mountains. Dr. Taft's work on the REGISTER necessarily took him to all dental society meetings and made him acquainted with the members of the profession all over the country.

With a few other noble men of the early days he conceived the idea of making his calling a profession, and he gave largely of his time and means to the organization of dental societies and conventions, for the open discussion of methods of practice and scientific investigation. In those days many things which are now commonly known were "trade secrets," and must be purchased by large sums of money or obtained by long service and under bonds of secrecy. He valued the convention method of education highly, and was ever ready to contribute to the proceedings of any such organization. He assisted in organizing all the national societies. He was a good writer on technical topics and a forcible speaker. He engaged in all the great debates on topics which sometimes threatened to wreck all the efforts that had been made to consolidate the profession and give it ethical standing. Dr. Taft always stood for high professional ideals and attainments.

In 1875 the Regents of the University of Michigan decided to add a department of Dentistry, and Dr. Taft was invited to take charge of the organization. He was, at that time, Professor of Operative Dentistry in the Ohio College of Dental Surgery and dean of that school, but accepted the call to Michigan on condition that he should give only part of his time to the work. He resigned the place of dean in the Ohio College at this time, but kept his professorship until 1878.

He took up the work at Michigan University not expecting to continue it permanently; but he saw the opportunity of developing here a system of dental instruction on a different basis from that which obtained in other places, and decided at a considerable sacrifice to continue with this institution, and, through it, try to elevate the standards of dental education. He sought to make available all the instructions given in other departments of the University that would extend the culture and increase the capacity of dental students for higher service. Through his efforts and the co-operation of the University authorities he had the course of study required for the degree extended from two years of six months to two years of nine months, and a few years later the course was again extended to three years of nine months, and in 1901 the last great advance to a four year's course was made. At the same time the entrance requirement was raised to a high-school graduation or its equivalent, making the highest standard of graduation in the country. The National Association of Dental Faculties in 1902 decided to raise the standard in all the schools in its membership to a four year's course of seven months, with admission standard of entrance to the third year in high school. It is not meant to say here that Dr, Taft is personally responsible for this advance in educational standards, but that he was an important personal element in creating a sentiment which demanded this standard, there can be no doubt. He kept his school well abreast of the highest standards advocated by the profession.

In 1884 he was largely instrumental in organizing the National Association of Dental Faculties. This body has done more to unify dental educational standards and advance them than all other organizations combined. He was greatly interested in the work of this body and attended every meeting ever held by it, and had filled all its offices with the exception of Secretary and Treasurer. He believed in the organization and hoped that it would be the means of arriving at a standard of qualification which would be accepted in every State, and perhaps in other countries.

He was a member of the Ohio and Michigan State and many local dental societies, and did much to make the meetings of these societies profitable to those attending. He read many papers before them and was always ready to participate in the discussions. He was so generally informed that scarcely any subject could be considered in these meetings that did not interest him. And he never got so absorbed in the literary and scientific topics that he was not eager to notice every new method or appliance for the advantage of technical practice. He was a remarkably broad man and his sympathies were easily reached by anything new or valuable pertaining to the theory or practice of dentistry.

He collected a valuable library of dental literature which in 1899 he sold to the North-Western Dental School of Chicago. Since that time he has with considerable effort collected another journal library and had succeeded so well that he had secured complete sets of the more important dental periodicals. By his suggestion the University of Michigan is in possession of one of the best dental libraries in the country.

As a member of the profession of dentistry he did everything he could to educate its members, and by his devotion to it he showed most clearly that it was the one object to which he gave his first and best efforts. He was a successful practitioner, judged from every standpoint, and freely used what he might have saved, for the advancement of the profession and in various forms of benevolence.

He took great interest in benevolent work of various kinds. He was an earnest member of the Congregational Church for many years, and served his church in many official relations. He was superintendent of the Vine Street Congregational Church Sabbath-school for twenty-five years. He wasRegistrar of Miami Conference for about thirty years. He was Assistant Superintendent of the great Bethel Sunday-School in Cincinnati, and a member of its Board of Directors for many years. Through his personal efforts a mission enterprise for mountain whites in the mountains of Kentucky was organized several years ago which has become an influence for good in all that part of the State. In many other ways he has shown his interest in the general welfare of the poor and helpless. He took a deep interest in political matters, while never personally engaged in active politics he supported men always who stood for high moral principles.

He was a man of indomitable will or purpose, but held his personal preferences so completely in check that he was always ready to comply with the plans of others, that united and harmonious effort might be had. He was kind and generous and charitable even to a fault, and was sometimes the victim of imposition. He was indeed a remarkable man, and he will be missed in many places. He will probably be missed from the dental conventions more than any other man. Few men in any calling have accomplished more than has Dr. Taft. The memory of his life will be an inspiration to many younger lives to make better use of their opportunities.

It was a touching tribute which the large number of his friends paid to him at the burial in a beautiful spot in Spring Grove Cemetery, at Cincinnati. Several of his professional associates have suggested that the profession should erect over his grave a suitable monument which should help to perpetuate his name. This would be a very happy and fitting memorial; but whether it shall be done or not his life has made an impression that will probably outlive any monument of stone. Death came to him suddenly, but in the fullness of time. It found him ready, but not waiting. He was at his post when stricken, doing work which other and younger men would hesitate to undertake.

JONATHAN TAFT, D.D.S., M.D.

## Figures and Tables

**Figure f1:**